# Dynamics Analysis and Simulation of a Modified HIV Infection Model with a Saturated Infection Rate

**DOI:** 10.1155/2014/145162

**Published:** 2014-03-23

**Authors:** Qilin Sun, Lequan Min

**Affiliations:** ^1^School of Automation and Electrical Engineering, University of Science and Technology Beijing, Beijing 100083, China; ^2^School of Mathematics and Physics, University of Science and Technology Beijing, Beijing 100083, China

## Abstract

This paper studies a modified human immunodeficiency virus (HIV) infection differential equation model with a saturated infection rate. It is proved that if the basic virus reproductive number *R*_0_ of the model is less than one, then the infection-free equilibrium point of the model is globally asymptotically stable; if *R*_0_ of the model is more than one, then the endemic infection equilibrium point of the model is globally asymptotically stable. Based on the clinical data from HIV drug resistance database of Stanford University, using the proposed model simulates the dynamics of the two groups of patients' anti-HIV infection treatment. The numerical simulation results are in agreement with the evolutions of the patients' HIV RNA levels. It can be assumed that if an HIV infected individual's basic virus reproductive number *R*_0_ < 1 then this person will recover automatically; if an antiretroviral therapy makes an HIV infected individual's *R*_0_ < 1, this person will be cured eventually; if an antiretroviral therapy fails to suppress an HIV infected individual's HIV RNA load to be of unpredictable level, the time that the patient's HIV RNA level has achieved the minimum value may be the starting time that drug resistance has appeared.

## 1. Introduction

The human immunodeficiency virus (HIV) mainly targets a host's CD4^+^ T cells. Chronic HIV infection causes gradual depletion of the CD4^+^ T cell pool and thus progressively compromises the hosts immune response to opportunistic infections, leading to Acquired Immunodeficiency Syndrome (AIDS) [1].

In recent years, there is much work done on HIV infection from different points of view, such as pathology [2], microbiology [3], and mathematics [4–7]. Mathematical models have become essential tools to make assumptions, suggest new experiments, or help easily explain complex processes [8]. The basic mathematical model widely used for studying the dynamics of HIV infection has the following form [4, 9]:

(1) $$\begin{array}{c} \dot{x}=\lambda -d_{1}x-k_{1}xv,\\ \dot{y}=k_{1}xv-d_{2}y,\\ \dot{v}=ay-d_{3}v, \end{array}$$

where *x*(*t*),  *y*(*t*), and *v*(*t*) are the number of uninfected cells, infected cells, and free virus, respectively. Uninfected cells are produced at a constant rate *λ*, die at rate *d*_1_ *x*, and become infected at rate *k*_1_ *xv*. Infected cells are produced at rate *k*_1_ *xv* and die at rate *d*_2_ *y*. Free virus is produced from infected cells at rate *ay* and dies at rate *d*_3_ *v*.

Equation (1) has a basic virus reproductive number *R*_0_ = *ak*_1_ *λ*/(*d*_1_ *d*_2_ *d*_3_). According to Nowak and Bangham [4], *R*_0_ is defined as the number of newly infected cells arising from any one infected cell; if *R*_0_ is smaller than 1, then in the beginning of the infection, each virus infected cell produces on average less than one newly infected cell. Thus, the infection cannot spread, and the system returns to the uninfected state; if *R*_0_ is larger than 1, then initially each virus infected cell produces on average more than one newly infected cell. The infected cell population will increase, whereas the uninfected cell population will decline and therefore provide less opportunity for the virus to infect new cells.

There is a discussion about the process of the HIV RNA transcribing into DNA: when an HIV enters a resting CD4^+^ T cell, the HIV RNA may not be completely reverse transcribed into DNA [10]. A proportion of resting infected cells can revert to the uninfected state before the viral genome is integrated into the genome of the lymphocyte [11].

Recently, some mathematical models of HIV infection have been proposed based on the assumption that a fraction of infected CD4^+^ T cells return to the uninfected class [12–14]. Srivastava and Chandra [13] have considered a model with three populations: uninfected CD4^+^ T cells (*x*), infected CD4^+^ T cells (*y*), and HIV population (*v*). The model has the following form:

(2) $$\begin{array}{c} \dot{x}=\lambda -d_{1}x-k_{1}xv+py,\\ \dot{y}=k_{1}xv-\left(d_{2}+p\right)y,\\ \dot{v}=ay-d_{3}v, \end{array}$$

where the meanings of the variables *x*(*t*), *y*(*t*), and *v*(*t*) and the parameters *λ*,  *d*_1_,  *k*_1_,  *d*_2_,  *a*, and *d*_3_ are the same as those given in (1). The term *py* is the rate of infected cells in the latent stage reverting to the uninfected class. Equation (2) also has a basic virus reproductive number *R*_0_ = *ak*_1_ *λ*/(*d*_3_ *d*_1_(*p* + *d*_2_)). They have proved that if *R*_0_ ≤ 1, the infection-free steady state of (2) is globally asymptotically stable; if *R*_0_ > 1, the endemic steady state of (2) is globally asymptotically stable [13].

In (2), the mass action term *k*_1_ *xv* used to model infection of CD4^+^ T cells by free virions is biologically problematic for several reasons. Firstly, since *λ*/*d*_1_ represents the total number of CD4^+^ T cells in the basic virus reproductive number *R*_0_ = *ak*_1_ *λ*/(*d*_3_ *d*_1_(*p* + *d*_2_)), this causes *R*_0_ to depend upon the total number of CD4^+^ T cells in vivo. This implies the dubious prediction that individuals with more CD4^+^ T cells will be more easily infected than individuals with less CD4^+^ T cells. Secondly, the rate of HIV infection is assumed to be bilinear by the mass action term *k*_1_ *xv*. However, the actual incidence rate is probably not linear over the entire range of virus *v*(*t*) and uninfected CD4^+^ T cells *x*(*t*) [15–17].

On biological grounds, during primary HIV infection, the rate of virus infection should be approximately proportionate to the virus load *k*_1_ *v* because of a small amount of viral load with respect to a large number of CD4^+^ T cells. However,since the total number of healthy CD4^+^ T cells in vivo is limited, the HIV infection will approach saturation with more and more virus produced. In this case, it is more reasonable to assume that the rate of virus infection should be approximately proportionate to the number of healthy CD4^+^ T cells *k*_1_ *x*.

Based on the argument above, this paper describes an amended model. In this model, we use a saturated infection rate *k*_1_ *xv*/(*x* + *v*) to replace the mass action term *k*_1_ *xv* in (2). Under the formulation of this saturated infection rate, the basic virus reproductive number *R*_0_ is independent of the total number of CD4^+^ T cells. Meanwhile, the actual incidence rate is not linear over the entire range of virus *v*(*t*) and uninfected CD4^+^ T cells *x*(*t*) any more. The global stabilities of the infection-free state and the endemic infection state of the modified HIV infection model have been discussed. Based on the clinical data from HIV drug resistance database of Stanford University, using the proposed model simulates the dynamics of two groups of patients' anti-HIV infection treatment, and then make long-term predictions for the two groups' anti-HIV infection treatment, respectively.

The rest of this paper is organized as follows.  Section 2 introduces a modified model and discusses the boundedness of the solutions of the model. Sections 3 and 4 discuss the global stability of the infection-free state and the endemic infection state of the modified HIV infection model, respectively.  Section 5 simulates the dynamics of two groups of patients' anti-HIV infection treatment.  Section 6 summarizes this paper.

## 2. Modified HIV Infection Model

### 2.1. The Modified HIV Infection Model

Based on (2), our modified HIV infection model has the following form:

(3) $$\begin{array}{c} \dot{x}=\lambda -d_{1}x-\frac{k_{1}xv}{x+v}+py,\\ \dot{y}=\frac{k_{1}xv}{x+v}-\left(d_{2}+p\right)y,\\ \dot{v}=ay-d_{3}v, \end{array}$$

where the meanings of the variables *x*(*t*), *y*(*t*), and *v*(*t*) and the parameters *λ*,  *d*_1_,  *k*_1_,  *d*_2_,  *p*,  *a*, and *d*_3_ are the same as those given in (2). Equation (3) has two steady states:

(1) The infection-free steady state
  (4) $$\begin{array}{c} Q_{1}=\left(x_{0},0,0\right) \end{array}$$
 represents the virus infection free. *Q*_1_ is called infection-free equilibrium point. Here,
  (5) $$\begin{array}{c} x_{0}=\frac{\lambda }{d_{1}}. \end{array}$$
(2) The endemic infected steady state
  (6) $$\begin{array}{c} Q_{2}=\left(\bar{x},\bar{y},\bar{v}\right) \end{array}$$
 represents persistent virus infection. *Q*_2_ is called endemic infection equilibrium point. Here,
  (7) $$\begin{array}{c} \bar{x}=\frac{\lambda R_{0}}{k_{1}\left(R_{0}-1\right)+d_{1}R_{0}-p\left(R_{0}-1\right)R_{0}\left(d_{3}/a\right)},\\ \bar{y}=\frac{d_{3}}{a}\left(R_{0}-1\right)\bar{x},\;\;\bar{v}=\frac{a\bar{y}}{d_{3}}=\left(R_{0}-1\right)\bar{x}. \end{array}$$
 Here,
  (8) $$\begin{array}{c} R_{0}=\frac{ak_{1}}{d_{3}\left(d_{2}+p\right)}. \end{array}$$

Since the total rate of disappearance of infected cells is *d*_2_ + *p*, infected cells live on average for time 1/(*d*_2_ + *p*). Each infected cell produces virus at rate *a*. Thus, each infected cell produces on average a total of *a*/(*d*_2_ + *p*) viruses. Since virus dies at rate *d*_3_ per virion, each virus survives on average for time 1/*d*_3_. During the time 1/*d*_3_, each virus infects on average *k*_1_ *x*_0_/(*x*_0_ + *v*_0_)*d*_3_ cells, where *x*_0_ and *v*_0_ are the preinfection target cells' density and viruses' density, respectively. Thus, the total number of cells infected by the *a*/(*d*_2_ + *p*) viruses is *ak*_1_ *x*_0_/(*x*_0_ + *v*_0_)*d*_3_(*d*_2_ + *p*). According to (4) and (5), *x*_0_ = *λ*/*d*_1_ and *v*_0_ = 0 at the preinfection steady state. Then one can obtain that the total number of cells infected by each infected cell is *ak*_1_/*d*_3_(*d*_2_ + *p*). Hence, *R*_0_ is the basic virus reproductive number of (3) which is independent of the total number of the uninfected CD4^+^ T cells.

According to (4), (6), and (7), if *R*_0_ ≤ 1, then *Q*_1_ is the unique infection-free equilibrium point; if *R*_0_ > 1, then, in addition to the infection-free equilibrium point, (3) has another equilibrium point *Q*_2_.

### 2.2. Boundedness of Solutions

It is easy to show that the solutions of (3) with initial conditions *x*(0) > 0,  *y*(0) > 0, and *v*(0) > 0 have all positive components for *t* > 0. Hence, one begins the analysis of (3) by observing the nonnegative octant

(9) $$\begin{array}{c} D=\left\{\left(x,y,v\right)\in R_{+}^{3}:x\geq 0,y\geq 0,v\geq 0\right\}. \end{array}$$

According to the first two equations of (3), one can get

(10) $$\begin{array}{c} \dot{x}+\dot{y}=\lambda -d_{1}x-d_{2}y\leq \lambda -d\left(x+y\right),\\ d=\min\left(d_{1},d_{2}\right), \end{array}$$

and then

(11) $$\begin{array}{c} x+y\leq \frac{\lambda }{d}. \end{array}$$

So *x*(*t*) and *y*(*t*) are bounded. From the last equation of (3), it follows that

(12) $$\begin{array}{c} \dot{v}=ay-d_{3}v\leq \frac{a\lambda }{d}-d_{3}v, \end{array}$$

and then

(13) $$\begin{array}{c} v\leq \frac{a\lambda }{dd_{3}}. \end{array}$$

So *v*(*t*) are bounded. Hence there is a bounded subset of *D*:

(14) $$\begin{array}{c} \Omega =\left\{\left(x,y,v\right)\in R_{+}^{3}:0\leq x+y\leq \frac{\lambda }{d},0\leq v\leq \frac{a\lambda }{dd_{3}}\right\} \end{array}$$

such that any solution trajectory (*x*(*t*), *y*(*t*), *v*(*t*)) of (3) with initial value (*x*(0),  *y*(0),  *v*(0)) in *Ω* will keep in the subset *Ω*.

According to (7), $\bar{x}>0$, $\bar{y}>0$, and $\bar{v}>0$. One can get that the endemic infection equilibrium point *Q*_2_ exists in the interior of *Ω*:

(15) Ω0={(x,y,v)∈Ω:0<x,0<y,0<x+y<λd, 0<v<aλdd3}.

Therefore, the stability of the endemic infection equilibrium point *Q*_2_ only needs to be discussed in *Ω* ^0^.

## 3. Stability of the Infection-Free Equilibrium Point *Q*_1_

In this section, we discuss locally asymptotical stability and globally asymptotical stability of the infection-free equilibrium point *Q*_1_ of (3).

### 3.1. Locally Asymptotical Stability of the Infection-Free**** Equilibrium Point *Q*_1_

**Theorem 1**

If *R*_0_ = *ak*_1_/(*d*_3_(*d*_2_ + *p*)) < 1, then the infection-free equilibrium point *Q*_1_ of (3) is locally asymptotically stable. If *R*_0_ > 1, then the infection-free equilibrium point *Q*_1_ is unstable.

**Proof**

The Jacobi matrix of (3) at an arbitrary point is given by

(16) $$\begin{array}{c} J\left(x,y,v\right)=\left[\begin{array}{ccc} -d_{1}-a_{1} & p & -a_{2}\\ a_{1} & -p-d_{2} & a_{2}\\ 0 & a & -d_{3} \end{array}\right], \end{array}$$

where *a*_1_ = *k*_1_ *v* ^2^/(*x* + *v*)^2^ and *a*_2_ = *k*_1_ *x* ^2^/(*x* + *v*)^2^.

Substituting the equilibrium point *Q*_1_ into matrix (16) gives

(17) $$\begin{array}{c} J_{Q_{1}}=\left[\begin{array}{ccc} -d_{1} & p & -k_{1}\\ 0 & -p-d_{2} & k_{1}\\ 0 & a & -d_{3} \end{array}\right]. \end{array}$$

The corresponding eigenequation is

(18) $$\begin{array}{c} \left|\lambda E-J_{Q_{1}}\right|=\left|\begin{array}{ccc} \lambda +d_{1} & -p & k_{1}\\ 0 & \lambda +p+d_{2} & -k_{1}\\ 0 & -a & \lambda +d_{3} \end{array}\right|=0. \end{array}$$

Solving

(19) $$\begin{array}{c} \left|\lambda E-J_{Q_{1}}\right|=\left(\lambda +d_{1}\right)\left[\left(\lambda +p+d_{2}\right)\left(\lambda +d_{3}\right)-ak_{1}\right]=0 \end{array}$$

gives

(20) $$\begin{array}{c} \lambda_{1}=-d_{1}<0, \end{array}$$

(21) $$\begin{array}{c} \left(\lambda +d_{2}+p\right)\left(\lambda +d_{3}\right)-ak_{1}k_{3}=0. \end{array}$$

Equation (21) can be written as

(22) $$\begin{array}{c} \lambda^{2}+\left(d_{2}+p+d_{3}\right)\lambda +d_{3}\left(p+d_{2}\right)-ak_{1}=0. \end{array}$$

Solving equation (22) gives

(23) λ2=−(d2+p+d3)−(d2+p+d3)2−4[d3(p+d2)−ak1]2=−(d2+p+d3)−(d2+p+d3)2−4d3(p+d2)(1−R0)2,λ3=−(d2+p+d3)+(d2+p+d3)2−4[d3(p+d2)−ak1]2=−(d2+p+d3)+(d2+p+d3)2−4d3(p+d2)(1−R0)2.

If *R*_0_ < 1, then *λ*_2_ < 0 and *λ*_3_ < 0. Hence the infection-free equilibrium point *Q*_1_ is locally asymptotically stable. If *R*_0_ > 1, then *λ*_3_ > 0 such that the infection-free equilibrium point *Q*_1_ is unstable.

### 3.2. Globally Asymptotical Stability of the Infection-Free**** Equilibrium Point *Q*_1_

**Theorem 2**

If *R*_0_ < 1, then the infection-free equilibrium point *Q*_1_ of (3) is globally asymptotically stable in *Ω*.

**Proof**

Define a global Lyapunov function by

(24) $$\begin{array}{c} V_{1}\left(x,y,v\right)=y+\frac{\left(d_{2}+p\right)v}{a}. \end{array}$$

The derivative of *V*_1_(*x*,  *y*,  *v*) along the positive solutions of (3) is

(25) V˙1=y˙+(d2+p)v˙a=k1xvx+v−d2y−py+(d2+p)y −d3(d2+p)va≤k1v−d3(d2+p)va=[ak1d3(d2+p)−1]d3(d2+p)av=(R0−1)d3(d2+p)av.

If *R*_0_ < 1, then ${\dot{V}}_{1}\leq 0$ holds in *Ω*. Moreover, ${\dot{V}}_{1}=0$ if and only if *v* = 0. Hence, the largest compact invariant set in *Ω* is

(26) $$\begin{array}{c} E_{1}=\left\{\left(x,y,v\right)\in \Omega \;|\;{\dot{V}}_{1}=0\right\}=\left\{\left(x,y,v\right)\in \Omega \;|\;v=0\right\}. \end{array}$$

According to the LaSalle's invariance principle, lim⁡_*t*→+*∞*_ *v*(*t*) = 0. Then one can get limit equations:

(27) $$\begin{array}{c} \dot{x}=\lambda -d_{1}x+py,\\ \dot{y}=-d_{2}y-py. \end{array}$$

Define a global Lyapunov function by

(28) $$\begin{array}{c} V_{2}\left(x,y\right)=x-x_{0}-x_{0}\ln\frac{x}{x_{0}}+y, \end{array}$$

where

(29) $$\begin{array}{c} \lambda =d_{1}x_{0},\;\;Q_{1}=\left(x_{0},0,0\right). \end{array}$$

The derivative of *V*_2_(*x*, *y*) along the positive solutions of (27) is

(30) V˙2=x˙+y˙−x0xx˙=λ−d1x+py−d2y−py−x0x(λ−d1x+py).

Since *λ* = *d*_1_ *x*_0_,

(31) V˙2=d1x0−d1x−x0xd1x0+d1x0−x0xpy−d2y=d1x0[2−xx0−x0x]−(x0xp+d2)y.

Since the arithmetic mean is greater than or equal to the geometric mean, we obtain 2 − (*x*/*x*_0_) − (*x*_0_/*x*) ≤ 0.

Therefore, ${\dot{V}}_{2}\leq 0$ holds in *E*_1_, and ${\dot{V}}_{2}=0$ if and only if *x* = *x*_0_ and *y* = 0. There is the largest compact invariant set in *E*_1_:

(32) E2={(x,y,v)∈E1 ∣ V˙2=0}={(x,y,v)∈E1 ∣ x=x0,y=0}={Q1}.

Hence if *R*_0_ < 1, all solution paths in *Ω* approach the infection-free equilibrium point *Q*_1_.

## 4. Stability of the Endemic Infection Equilibrium Point *Q*_2_

In this section, we analyze local asymptotical stability and global asymptotical stability of the endemic infection equilibrium point *Q*_2_ of (3).

### 4.1. Locally Asymptotical Stability of the Endemic Infection Equilibrium Point *Q*_2_

**Theorem 3**

If *R*_0_ > 1, then the endemic infection equilibrium point *Q*_2_ of (3) is locally asymptotically stable.

**Proof**

Put the equilibrium point *Q*_2_ into matrix (16); then one obtains

(33) JQ2=[−d1−k1v¯2(x¯+v¯)2p−k1x¯2(x¯+v¯)2k1v¯2(x¯+v¯)2−p−d2k1x¯2(x¯+v¯)20a−d3],|λE−JQ2|=|λ+d1+k1v¯2(x¯+v¯)2−pk1x¯2(x¯+v¯)2−k1v¯2(x¯+v¯)2λ+p+d2−k1x¯2(x¯+v¯)20−aλ+d3|=0.

Solving the eigenequation of the matrix above, here is

(34) λ3  +[d1+d2+d3+p+k1v¯2(x¯+v¯)2]︸a1λ2+[d3(d2+p)+(d1+k1v¯2(x¯+v¯)2)(d2+p+d3)−pk1v¯2(x¯+v¯)2−ak1x¯2(x¯+v¯)2]︸a2λ+d3(d2+p)(d1+k1v¯2(x¯+v¯)2)+ak12x¯2v¯2(x¯+v¯)2−pd3k1v¯2(x¯+v¯)2−(d1+k1v¯2(x¯+v¯)2)ak1x¯2(x¯+v¯)2︸a3=0.

If *R*_0_ > 1, one obtains that

(35) a1=d1+d2+d3+p+k1v¯2(x¯+v¯)2>0,a2=d3(d2+p)+(d1+k1v¯2(x¯+v¯)2)(d2+p+d3) −pk1v¯2(x¯+v¯)2−ak1x¯2(x¯+v¯)2=ak1R0+d1(d2+d3+p) +k1v¯2(x¯+v¯)2(d2+d3)−ak1x¯2(x¯+v¯)2=ak1R0−ak1x¯2R02x¯2+d1(d2+d3+p) +k1v¯2(x¯+v¯)2(d2+d3)=ak1(R0−1)R02+d1(d2+d3+p) +k1v¯2(x¯+v¯)2(d2+d3)>0,a3=d3(d2+p)(d1+k1v¯2(x¯+v¯)2) +ak12x¯2v¯2(x¯+v¯)2−pd3k1v¯2(x¯+v¯)2 −(d1+k1v¯2(x¯+v¯)2)ak1x¯2(x¯+v¯)2=d1d3(d2+p)−ad1k1x¯2(x¯+v¯)2+d2d3k1v¯2(x¯+v¯)2=d1ak1(R0−1)R02+d2d3k1v¯2(x¯+v¯)2>0,a1a2−a3=(d2+d3+p+k1v¯2(x¯+v¯)2) ×ak1(R0−1)R02+d1(d2+d3+p) ×(d2+d3+p+d1+k1v¯2(x¯+v¯)2) +d2k1v¯2(x¯+v¯)2(d2+d3+p+d1+k1v¯2(x¯+v¯)2) +d3k1v¯2(x¯+v¯)2(d3+p+d1+k1v¯2(x¯+v¯)2)>0.

Hence all inequalities of the Routh-Hurwitz criterion are satisfied. Therefore, the endemic infection equilibrium point *Q*_2_ is locally asymptotically stable.

### 4.2. Globally Asymptotical Stability of the Endemic Infection Equilibrium Point *Q*_2_

In this subsection, we firstly introduce a lemma outlined by Li and Wang [18], and then using this lemma discusses the globally asymptotical stability of the endemic infection equilibrium point *Q*_2_ of (3).

The lemma is briefly summarized as follows.

Let *x* ↦ *f*(*x*) ∈ *R* ^*n*^ be a *C* ^1^ function for *x* in an open set Γ ⊂ *R* ^*n*^. Consider the differential system

(36) $$\begin{array}{c} \dot{x}=f\left(x\right). \end{array}$$

Denote by *x*(*t*, *x* ^0^) the solution to (36) such that *x*(0, *x* ^0^) = *x* ^0^. Let $\tilde{x}$ be an equilibrium point of (36). Li and Wang [18] made the following two basic assumptions:

(*H*_1_) there exists a compact absorbing set *K* ⊂ Γ;
(*H*_2_) equation (36) has a unique equilibrium $\tilde{x}$ in Γ.

Li and Wang (see Theorem  2.5 in [18]) have given the following lemma.

**Lemma (see [18])**

Assume that

(1) assumptions (*H*_1_) and (*H*_2_) hold;
(2) equation (36) satisfies the Poincaré-Bendixson Property;
(3) for each periodic solution *x* = *p*(*t*) to (36) with *p*(0) ∈ Γ, the linear system (the second additive compound system)
  (37) $$\begin{array}{c} \dot{w}\left(t\right)=\frac{\partial f^{\left[2\right]}}{\partial x}\left(P\left(t\right)\right)w\left(t\right) \end{array}$$
 is asymptotically stable, where ∂*f* ^[2]^/∂*x* is the second additive compound matrix of the Jacobian matrix ∂*f*/∂*x*;
(4) ${\left(-1\right)}^{n}\text{ det }(\left(\partial f/\partial x\right)(\tilde{x}))>0$.
 Then the unique equilibrium $\tilde{x}$ is globally asymptotically stable in Γ.

Now one uses Lemma 4 to show the following.

**Theorem 5**

If *R*_0_ > 1, then the endemic infection equilibrium point *Q*_2_ of (3) is globally asymptotically stable in *Ω* ^0^, where *Ω* ^0^ is defined by (15).

**Proof**

Based on Lemma 4, the proof of Theorem 5 has been implemented via the following four steps.

(1) For epidemic models and many other biological models where the feasible region is a bounded cone, (*H*_1_) is equivalent to the uniform persistence of the system [19]. By (15), *Ω* ^0^ is bounded, so it only needs to show the uniform persistence of (3). According to Proposition  3.3 in [20], the necessary and sufficient condition for the uniform persistence of (3) is equivalent to the equilibrium point *Q*_1_ being unstable.  Theorem 1 has shown that *Q*_1_ is unstable if *R*_0_ > 1. Therefore, (3) is uniformly persistent if *R*_0_ > 1 so that (*H*_1_) holds if *R*_0_ > 1.

Meanwhile, *Q*_1_ = (*x*_0_, 0,0) by (4), so *Q*_1_ does not exist in *Ω* ^0^. Hence, *Q*_2_ is the unique equilibrium point of (3) in *Ω* ^0^ so that (*H*_2_) holds.

The results above verify the condition (1) of Lemma 4.

(2) The Jacobian matrix of (3) is

(38) $$\begin{array}{c} J\left(x,y,v\right)=\left[\begin{array}{ccc} -d_{1}-a_{1} & p & -a_{2}\\ a_{1} & -p-d_{2} & a_{2}\\ 0 & a & -d_{3} \end{array}\right], \end{array}$$

where *a*_1_ = (*k*_1_ *v* ^2^/(*x* + *v*)^2^) and *a*_2_ = (*k*_1_ *x* ^2^/(*x* + *v*)^2^).

If *H* = diag⁡(1, −1,1), then

(39) $$\begin{array}{c} HJH=\left[\begin{array}{ccc} -d_{1}-\frac{k_{1}v^{2}}{x+v} & -p & -\frac{k_{1}x^{2}}{{\left(x+v\right)}^{2}}\\ -\frac{k_{1}v^{2}}{{\left(x+v\right)}^{2}} & -p-d_{2} & -\frac{k_{1}x^{2}}{{\left(x+v\right)}^{2}}\\ 0 & -a & -d_{3} \end{array}\right], \end{array}$$

and one can obtain that *HJH* has nonpositive off-diagonal elements in *Ω* ^0^. Therefore (3) is competitive in *Ω* ^0^. It is known that 3-dimensional competitive systems have the Poincaré-Bendixson Property [21]. Hence, (3) satisfies the Poincaré-Bendixson Property. This verifies condition (2) of Lemma 4.

(3) Let *P*(*t*) = (*x*(*t*), *y*(*t*), *v*(*t*)) be a periodic solution in *Ω* ^0^.

According to [22], if *B* = (*b*_*ij*_) is a 3 × 3 matrix, then the second additive compound *B* ^[2]^ of *B* is

(40) $$\begin{array}{c} B^{\left[2\right]}=\left[\begin{array}{ccc} b_{11}+b_{22} & b_{23} & -b_{13}\\ b_{32} & b_{11}+b_{33} & b_{12}\\ -b_{31} & b_{21} & b_{22}+b_{33} \end{array}\right]. \end{array}$$

The Jacobian matrix of (3) is

(41) $$\begin{array}{c} J\left(x,y,v\right)=\left[\begin{array}{ccc} -d_{1}-a_{1} & p & -a_{2}\\ a_{1} & -p-d_{2} & a_{2}\\ 0 & a & -d_{3} \end{array}\right], \end{array}$$

where *a*_1_ = *k*_1_ *v* ^2^/(*x* + *v*)^2^ and *a*_2_ = *k*_1_ *x* ^2^/(*x* + *v*)^2^.

And then the second additive compound matrix of the Jacobian matrix of (3) is given by

(42) $$\begin{array}{c} J^{\left[2\right]}=\left[\begin{array}{ccc} -d_{1}-\frac{k_{1}v^{2}}{{\left(x+v\right)}^{2}}-p-d_{2} & \frac{k_{1}x^{2}}{{\left(x+v\right)}^{2}} & \frac{k_{1}x^{2}}{{\left(x+v\right)}^{2}}\\ a & -d_{1}-\frac{k_{1}v^{2}}{{\left(x+v\right)}^{2}}-d_{3} & p\\ 0 & \frac{k_{1}v^{2}}{{\left(x+v\right)}^{2}} & -p-d_{2}-d_{3} \end{array}\right], \end{array}$$

and the second additive compound system of (3) along the periodic solution *P*(*t*) = (*x*(*t*), *y*(*t*), *v*(*t*)) is

(43) w˙1=(−d1−k1v2(x+v)2−p−d2)w1+k1x2(x+v)2w2+k1x2(x+v)2w3,w˙2=aw1+(−d1−k1v2(x+v)2−d3)w2+pw3,w˙3=k1v2(x+v)2w2+(−p−d2−d3)w3.

Define a global Lyapunov function by

(44) $$\begin{array}{c} V\left(w_{1},w_{2},w_{3},P\right)=||\left(w_{1},\frac{y\left(t\right)}{v\left(t\right)}w_{2},\frac{y\left(t\right)}{v\left(t\right)}w_{3}\right)||, \end{array}$$

where ||·|| is the norm in set *D* defined by

(45) $$\begin{array}{c} ||\left(w_{1},w_{2},w_{3}\right)||=\sup\left\{\left|w_{1}\right|,\left|w_{2}\right|+\left|w_{3}\right|\right\}. \end{array}$$

Suppose that the solution *P*(*t*) is periodic of least period *ω* > 0 and that *P*(0) ∈ *Ω* ^0^. According to [23], (3) is uniformly persistent, if there exists a positive constant *μ* such that

(46) lim⁡ inf⁡t→∞ x(t)≥μ,  lim⁡ inf⁡t→∞y(t)≥μ,lim⁡ inf⁡t→∞ v(t)≥μ.

Step (1) has shown that (3) is uniformly persistent if *R*_0_ > 1. Hence, there always exists a positive constant *μ* which satisfies (46). The orbit of *P*(*t*) remains at a positive distance from the boundary of *Ω* by the uniform persistence, and one can obtain that

(47) $$\begin{array}{c} y\left(t\right)\geq \mu ,\;\;v\left(t\right)\geq \mu \;\text{for}\;\text{large}\;\text{enough}\;t. \end{array}$$

Since *v* < *aλ*/(*dd*_3_) by (15),

(48) $$\begin{array}{c} V\left(w_{1},w_{2},w_{3},P\right)\geq \frac{\mu dd_{3}}{\text{a}\lambda }||\left(w_{1},w_{2},w_{3}\right)||, \end{array}$$

for all (*w*_1_, *w*_2_, *w*_3_) ∈ *R* ^3^.

Along a solution (*w*_1_, *w*_2_, *w*_3_) of (43), *V*(*w*_1_, *w*_2_, *w*_3_, *P*) becomes

(49) $$\begin{array}{c} V\left(w_{1},w_{2},w_{3},P\right)=\sup\left\{\left|w_{1}\right|,\frac{y\left(t\right)}{v\left(t\right)}\left(\left|w_{2}\right|+\left|w_{3}\right|\right)\right\}. \end{array}$$

The right-hand derivative of *V*(*t*) along the positive solution of (43) is

(50) D+|w1|≤(−d1−k1v2(x+v)2−p−d2)|w1|+k1x2(x+v)2(|w2|+|w3|),D+|w2|≤a|w1|+(−d1−k1v2(x+v)2−d3)|w2|+p|w3|D+|w3|≤k1v2(x+v)2|w2|+(−p−d2−d3)|w3|.

Therefore

(51) D+yv(|w2|+|w3|) =y˙v−yv˙v2(|w2|+|w3|)+yvD+(|w2|+|w3|) ≤yv(y˙y−v˙v)(|w2|+|w3|)  +yv(a|w1|+(−d1−d3)|w2|+(−d2−d3)|w3|) ≤ayv|w1|+yv(|w2|+|w3|)  ×(y˙y−v˙v−d3−min⁡(d1,d2)),

(52) $$\begin{array}{c} D_{+}V\left(t\right)\leq \sup\left\{g_{1}\left(t\right),g_{2}\left(t\right)\right\}V\left(t\right), \end{array}$$

where

(53) g1(t)=−d1−p−d2−k1v2(x+v)2+k1vx2y(x+v)2≤y˙y−d1,g2(t)=ayv+y˙y−v˙v−d3−min⁡(d1,d2)=y˙y−min⁡(d1,d2).

Denote *d* = min⁡(*d*_1_, *d*_2_), and then

(54) $$\begin{array}{c} \sup\left\{g_{1}\left(t\right),g_{2}\left(t\right)\right\}\leq \frac{\dot{y}}{y}-d. \end{array}$$

By (52) and Gronwall's inequality, one obtains

(55) $$\begin{array}{c} V\left(t\right)\leq V\left(0\right)y\left(t\right)e^{-dt}\leq \frac{V\left(0\right)e^{-dt}\lambda }{d}. \end{array}$$

*V*(*t*) → 0 when *t* → *∞*, and then (*w*_1_, *w*_2_, *w*_3_) → 0 when *t* → *∞* by (48). The second additive compound system is asymptotically stable. This verifies the condition (3) of Lemma 4.

(4) The Jacobi matrix of (3) at the endemic infection equilibrium *Q*_2_ is

(56) $$\begin{array}{c} J_{Q_{2}}=\left[\begin{array}{ccc} -d_{1}-\frac{k_{1}{\bar{v}}^{2}}{{\left(\bar{x}+\bar{v}\right)}^{2}} & p & -\frac{k_{1}{\bar{x}}^{2}}{{\left(\bar{x}+\bar{v}\right)}^{2}}\\ \frac{k_{1}{\bar{v}}^{2}}{{\left(\bar{x}+\bar{v}\right)}^{2}} & -p-d_{2} & \frac{k_{1}{\bar{x}}^{2}}{{\left(\bar{x}+\bar{v}\right)}^{2}}\\ 0 & a & -d_{3} \end{array}\right], \end{array}$$

and then

(57) det⁡(JQ2)=|−d1−k1v¯2(x¯+v¯)2p−k1x¯2(x¯+v¯)2k1v¯2(x¯+v¯)2−p−d2k1x¯2(x¯+v¯)20a−d3|=−[d1+k1v¯2(x¯+v¯)2](d2+p)d3−ak12x¯2v¯2(x¯+v¯)4 +d3pk1v¯2(x¯+v¯)2+ak1x¯2(x¯+v¯)2[d1+k1v¯2(x¯+v¯)2]=−d1(d2+p)d3−k1v¯2(x¯+v¯)2(d2+p)d3 −ak12x¯2v¯2(x¯+v¯)4+d3pk1v¯2(x¯+v¯)2+ad1k1x¯2(x¯+v¯)2+ak12x¯2v¯2(x¯+v¯)4=d1[ak1x¯2(x¯+v¯)2−(d2+p)d3]−d2d3k1v¯2(x¯+v¯)2 −pd3k1v¯2(x¯+v¯)2+d3pk1v¯2(x¯+v¯)2=ad1k1[(x¯x¯+v¯)2−1R0]−d2d3k1v¯2(x¯+v¯)2.

According to (7), $\bar{v}=(R_{0}-1)\bar{x}$, and then one can obtain

(58) det⁡(JQ2)=ad1k1[1R02−1R0]−d2d3k1v¯2(x¯+v¯)2=ad1k1(1−R0)R02−d2d3k1v¯2(x¯+v¯)2.

Since *J*_*Q*_2__ is a 3 × 3 matrix, one gets *n* = 3. Then

(59) $$\begin{array}{c} {\left(-1\right)}^{3}\det\left(J_{Q_{2}}\right)=-\frac{ad_{1}k_{1}\left(1-R_{0}\right)}{R_{0}^{2}}+\frac{d_{2}d_{3}k_{1}{\bar{v}}^{2}}{{\left(\bar{x}+\bar{v}\right)}^{2}}. \end{array}$$

If *R*_0_ > 1, then (−1)^3^det⁡(*J*_*Q*_2__) > 0 holds in *Ω* ^0^. This verifies condition (4) of Lemma 4.

Hence, if *R*_0_ > 1, then the endemic infection equilibrium point *Q*_2_ is globally asymptotically stable in *Ω* ^0^ by Lemma 4.

## 5. Numerical Simulation

In the first subsection, we determine some parameter values of an anti-HIV infection treatment model based on (3). In the second subsection, using the anti-HIV infection treatment model simulates the dynamics of the Group I's anti-HIV infection treatment. In the third subsection, using the anti-HIV infection treatment model simulates the dynamics of the Group II's anti-HIV infection treatment. In the fourth subsection, we make long-term predictions for the two groups' anti-HIV infection treatment, respectively.

### 5.1. Modeling

Baxter et al. [24] have reported a randomized study of antiretroviral management based on plasma genotypic antiretroviral resistance testing in HIV patients failing therapy, which was enrolled from 14 units of the Terry Beirn Community Programs for Clinical Research on AIDS and the Walter Reed Army Medical Center (see the HIV drug resistance database of Stanford University [25]). These patients were failing virologically on a combination antiretroviral regimen containing protease inhibitors (PI) and nucleoside reverse transcriptase inhibitors (NRTI) [24]. The patients were seen at 4, 8, and 12 weeks. At each follow-up visit, changes in antiretroviral treatment were recorded and the tested items included patients' plasma CD4^+^ T cells counts and plasma HIV-1 RNA levels by the Chiron 2.0 bDNA assay [24].

In the following subsections, we select, from [24, 25], two group patients' mean uninfected CD4^+^ T cells counts and mean HIV RNA levels to simulate and make long-term predictions for the patients' treatment outcomes. Group I consists of 15 patients. Group II consists of 13 patients. The two groups of patients received the same PI: ritonavir (RTV) and saquinavir (SQV). Additionally, Group I received NRTI: strvudine (D4T). Group II received NRTI: strvudine (D4T) and dideoxyinosine (DDI) [24, 25].

Based on (3), the anti-HIV infection treatment model has the form

(60) $$\begin{array}{c} \dot{x}=\lambda -d_{1}x-\frac{\left(1-m\right)k_{1}vx}{x+v}+py,\\ \dot{y}=\frac{\left(1-m\right)k_{1}vx}{x+v}-d_{2}y-py,\\ \dot{v}=\left(1-n\right)ay-d_{3}v, \end{array}$$

where *m*, *n*  (0 ≤ *m*, *n* ≤ 1) are the efficacy variables of the treatment.

The infection-free equilibrium point *Q*_1_ of (60) is the same as that defined by (4):

(61) $$\begin{array}{c} Q_{1}=\left(\frac{\lambda }{d_{1}},0,0\right). \end{array}$$

The endemic infection equilibrium point *Q*_2_ of (60) is given by

(62) $$\begin{array}{c} Q_{2}=\left(\bar{x},\bar{y},\bar{v}\right), \end{array}$$

where,

(63) $$\begin{array}{c} \bar{x}=\frac{\lambda R_{0}}{\left(1-m\right)k_{1}\left(R_{0}-1\right)+d_{1}R_{0}-p\left(R_{0}-1\right)R_{0}\left(d_{3}/\left(1-n\right)a\right)},\\ \bar{y}=\frac{d_{3}}{\left(1-n\right)a}\left(R_{0}-1\right)\bar{x},\;\;\bar{v}=\frac{\left(1-n\right)a\bar{y}}{d_{3}}=\left(R_{0}-1\right)\bar{x}, \end{array}$$

and *R*_0_ is the basic virus reproductive number of (60):

(64) $$\begin{array}{c} R_{0}=\frac{\left(1-n\right)\left(1-m\right)ak_{1}}{d_{3}\left(d_{2}+p\right)}. \end{array}$$

Determine the parameter value ranges of (60).

(1) Naive CD4^+^ T cells decayed with an average half-life of 50 days [26]. Therefore, one obtains
  (65) $$\begin{array}{c} d_{1}=\frac{-\ln\left(0.5\right)}{50}. \end{array}$$
 Hence *d*_1_ ≈ 0.0139. Because the apoptosis of CD4^+^ T cells is raised by HIV infection [27, 28], *d*_1_ should be more than 0.0139 during the simulation.
(2) Since healthy individuals have an average of 830/*μ*L CD4^+^ T cells [29],
  (66) $$\begin{array}{c} \lambda =d_{1}\times 830\approx 0.0139\times 830=11.5370. \end{array}$$
(3) Since the cells that produce the virus are also short-lived, with a half-life of approximately 1.2 days [30], one obtains
  (67) $$\begin{array}{c} d_{2}=\frac{-\ln\left(0.5\right)}{1.2}\approx 0.5776. \end{array}$$
(4) Since the half-life of HIV-1 in the plasma appears to be only 1 to 2 days [30], one selects
  (68) $$\begin{array}{c} d_{3}=\frac{-\ln\left(0.5\right)}{1.5}\approx 0.4621. \end{array}$$
(5) Because only a small fraction of cells in the eclipse phase will revert to the uninfected state, it assumes that *p* = 0.01 [12]. Hence one obtains
  (69) $$\begin{array}{c} p=0.01. \end{array}$$
(6) According to reference [31], one can determine the other parameter value ranges as follows:
  (70) $$\begin{array}{c} k_{1}\in \left[2.5\times 10^{-5},0.5\right],\;\;a\in \left[2,1250\right],\\ m\in \left[0,1\right],\;n\in \left[0,1\right]. \end{array}$$

In each group, there was one patient whose clinical data was not complete. Therefore we do not conclude the two patients' clinical data in the following simulations.

### 5.2. The Mean Dynamics Simulation of Group I's Anti-HIV Infection Treatment

Using the other 14 patients' clinical data determines the equation parameter values as follows:

(71) $$\begin{array}{c} k_{1}=6\times 10^{-2},\;\;a=37. \end{array}$$

The value changes of the parameters *d*_1_, *m*, and *n* and the basic virus reproductive number *R*_0_ are listed in Table 1.

**Figure 1:**
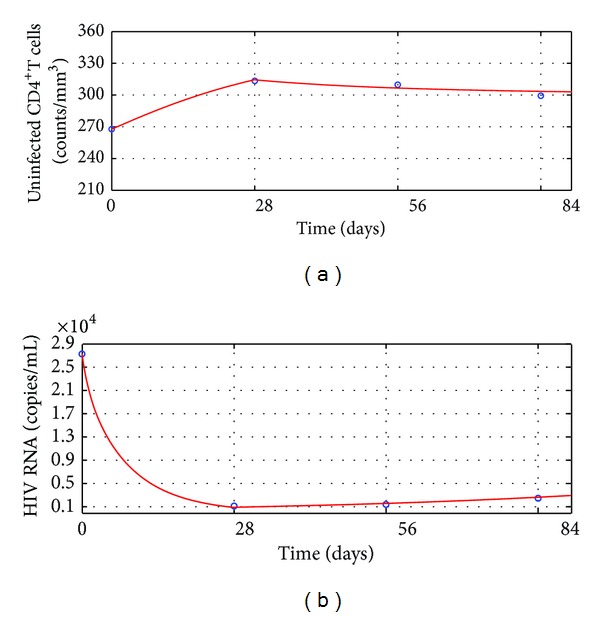
Outcomes of the treatment efficacy of Group I. Circles: the clinical data; solid line: the numerical simulation of (60). (a) Mean uninfected CD4^+^ T cells counts. (b) Mean HIV RNA levels.

The simulation results of the mean dynamics of anti-HIV infection treatment of Group I are shown in Figure 1. During the first 4 weeks, the treatment reduced the basic virus reproductive number *R*_0_ from 8.1759 to 0.6148. Hence the patients' mean HIV RNA levels decreased rapidly to approach infection-free steady state *Q*_1_ defined by (4) as Theorem 2 predicts.

However, after the 4th week, the resistance to antiretroviral drugs appeared. It made the therapy efficacy parameters *m* and *n* decrease from 0.53 to 0.45 and 0.84 to 0.76, respectively. Meanwhile the apoptosis of CD4^+^ T cells was raised by HIV more strongly. Hence *d*_1_ rose from 0.033 to 0.038. The suboptimal treatment increased *R*_0_ value of Group I from 0.6148 to 1.0792. As a result, the patients' mean HIV RNA levels increased slowly to converge to a new infected steady state *Q*_2_ defined by (62) and (63) as Theorem 5 predicts. On the other hand, observe that the mean uninfected CD4^+^ T cell counts of Group I increased rapidly in the first 4 weeks and decreased slowly in the following 8 weeks.

### 5.3. The Mean Dynamics Simulation of Group II's Anti-HIV Infection Treatment

Using 12 patients' clinical data determines the equation parameter values as follows:

(72) $$\begin{array}{c} k_{1}=6\times 10^{-2},\;\;a=26. \end{array}$$

The value changes of the parameters *d*_1_, *m*, and *n* and the basic virus reproductive number *R*_0_ are listed are listed in Table 2.

**Figure 2:**
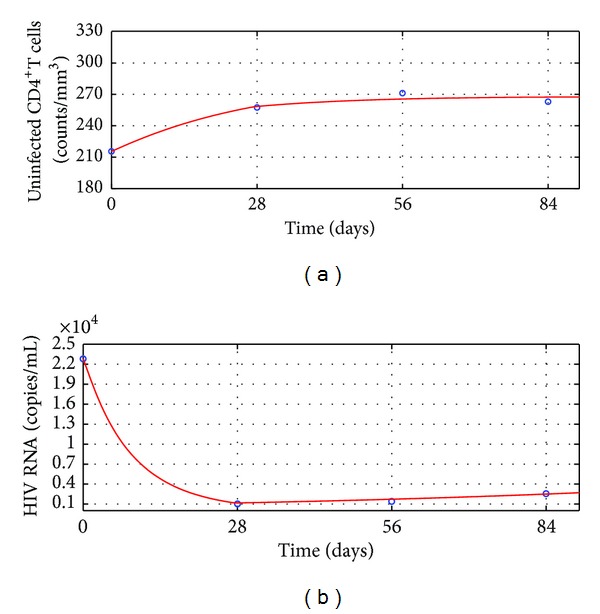
Outcomes of the treatment efficacy of Group II. Circles: the clinical data; solid line: the numerical simulation of (60). (a) Mean uninfected CD4^+^ T cells counts. (b) Mean HIV RNA levels.

The simulation results of the mean dynamics of anti-HIV infection treatment of Group II are shown in Figure 2. During the first 4 weeks, the treatment reduced the basic virus reproductive number *R*_0_ from 5.7452 to 0.6435. Hence the patients' mean HIV RNA levels decreased rapidly to approach to infection-free steady state *Q*_1_ defined by (4) as Theorem 2 predicts.

However, after the 4th week, the resistance to antiretroviral drugs appeared. It made the therapy efficacy parameters *m* and *n* decrease from 0.65 to 0.55 and 0.68 to 0.59, respectively. Meanwhile the apoptosis of CD4^+^ T cells was raised by HIV more strongly. Hence *d*_1_ rose from 0.041 to 0.0428. The suboptimal treatment made *R*_0_ value of Group II increase from 0.6435 to 1.0600. As a result, the patients' mean HIV RNA levels increased slowly to converge to a new infected steady state *Q*_2_ defined by (62) and (63) as Theorem 5 predicts. On the other hand, observe that the mean uninfected CD4^+^ T cell counts of Group II increased rapidly in the first 4 weeks but rose slowly in the following 8 weeks.

### 5.4. The Long-Term Predictions for the Two Groups' Anti-HIV Infection Treatment

According to 2013 HIV therapy guidelines published by World Health Organization (WHO) [32], viral load is recommended as the preferred monitoring approach to diagnose and confirm antiretroviral treatment failure; treatment failure is defined by a persistently detectable viral load exceeding 1000 copies/mL after at least six months of using antiretroviral drugs. However, HIV RNA levels of the two groups of patients were only tested at 4, 8, and 12 weeks in the study [24]. Therefore, it is necessary to make long-term predictions to detect whether the treatments for the two groups are failure.

**Figure 3:**
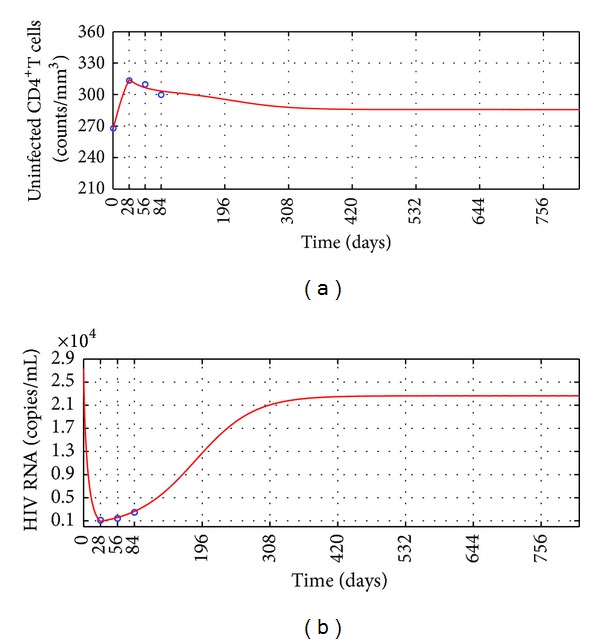
The long-term prediction for the treatment efficacy of Group I. Circles: the clinical data; solid line: the numerical simulation of (60). (a) Mean uninfected CD4^+^ T cells counts. (b) Mean HIV RNA levels.

**Figure 4:**
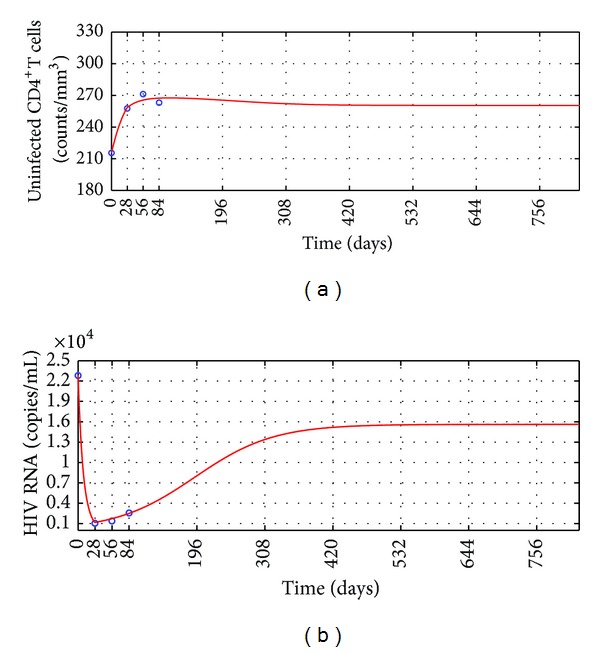
The long-term prediction for the treatment efficacy of Group II. Circles: the clinical data; solid line: the numerical simulation of (60). (a) Mean uninfected CD4^+^ T cells counts. (b) Mean HIV RNA levels.

Assume that after ending the 12 weeks' antiretroviral treatment testing, the two groups keep receiving the anti-HIV infection treatment for 2 years. During the 2 years, the drug resistance does not become worse and all parameter values do not change. Using the numerical simulation of Equation (60) makes the long-term predictions for the two groups' anti-HIV infection treatment. The long-term prediction for Group I' anti-HIV infection treatment is shown in Figure 3. Two years' outcomes of the therapy for Group II are shown in Figure 4. Observe that after finishing the 12 weeks' antiretroviral treatment testing, the mean uninfected CD4^+^ T cells counts of the two groups both decline a little and finally keep at a level larger than the mean baseline values; the mean HIV RNA levels of the two groups both rise a lot to a level less than the mean baseline values but keep exceeding 1000 copies/mL all the time. The long-term predictions suggest that the treatments for the two groups are failure and better anti-HIV infection therapies should be considered.

## 6. Conclusion

This paper introduces a modified HIV infection differential equation model with a saturated infection rate *k*_1_ *xv*/(*x* + *v*) and the proportion of infected cells reverting to the uninfected state.

The basic virus reproductive number *R*_0_ of the model is independent of a patient's plasma total CD4^+^ T cell counts *λ*/*d*_1_, and the actual incidence rate is not linear over the entire range of virus *v*(*t*) and uninfected CD4^+^ T cells *x*(*t*) any more. This suggests that our model is more reasonable than the model proposed by [13].

The modified model has two equilibrium points: infection-free equilibrium point *Q*_1_ and endemic infection equilibrium point *Q*_2_. This paper discusses the locally asymptotical stabilities and globally asymptotical stabilities of the two equilibrium points, simulates the dynamics of two group patients' anti-HIV infection treatment, and makes long-term predictions for the two groups' anti-HIV infection treatment.

The theoretical results suggest the following.

1. If the basic virus reproductive number *R*_0_ < 1, then the infection-free equilibrium point *Q*_1_ of (3) is globally asymptotically stable in *Ω*. This means that if a person with *R*_0_ < 1, the person can recover automatically even if infected with a large amount of HIV; if a treatment makes a patient's *R*_0_ < 1, the patient will be cured eventually even if infected with a large amount of HIV.
2. The recent reports on three HIV infected patients have shown that some HIV infected patients may be cured via bone marrow transplants. The Berlin Patient was the first person cured of HIV [33]. After the Berlin Man, two cases reported cured of HIV in Kenya [34]. These reports can make one postulate that most individuals who connect HIV virus will not be infected by it and are not infecting other people. They will recover automatically without any treatment. The fact has not been well recognized since AIDS has been discovered in 1983. Mathematically, such phenomena can be described also via (3) where *k*_1_ = *a* = 0, and thus *R*_0_ = 0.
3. If the basic virus reproductive number *R*_0_ > 1, then the endemic infection equilibrium point *Q*_2_ of (3) is globally asymptotically stable in the interior of *Ω*. This means that a person with *R*_0_ > 1 will have endemic infection even if infected with only one HIV; if a treatment cannot make a patient's *R*_0_ < 1, the patient's HIV RNA in vivo cannot be cleared up eventually.

Based on the simulation results, one can propose the following hypotheses.

1. For a poor HIV treatment response patient, the drug resistance appears when the patient's HIV RNA level reduces to the first lowest level.
2. This hypothesis may interpret why the two group patients' mean CD4^+^ T cells counts rose and mean HIV RNA levels declined rapidly in the first 4 weeks but contrary in the following weeks (see Tables 1 and 2 and Figures 1 and 2).
3. Once a patient's drug resistance appears, the patient's HIV in vivo promotes the apoptosis of CD4^+^ T cells more strongly.
4. This hypothesis may interpret why between 4th week and 8th week the mean HIV RNA levels of the two groups kept a lower level than the first 4 weeks, but Group I's mean CD4^+^ T cells counts started to decrease slowly and Group II's mean CD4^+^ T cells counts rose more slowly than before (see Tables 1 and 2 and Figures 1 and 2).
5. According to the 2013 HIV therapy guidelines published by WHO [32], treatment failure is defined by a persistently detectable viral load exceeding 1000 copies/mL after at least six months of using antiretroviral drugs. Our long-term numerical simulation predictions suggest that after ending the 12 weeks' antiretroviral treatment [24], the two group patients' mean HIV RNA levels keep exceeding 1000 copies/mL during the additional 2 years' anti-HIV infection treatment. The treatments for the two groups are failure and better anti-HIV infection therapies should be considered. This means that the additional 2 years' treatments are not able to make patients obtain better outcomes. This may interpret why WHO defines that half a year's treatment cannot suppress a patient's HIV level below 1000 copies/mL to be treatment failure.

## Highlights

1. This paper introduces a modified HIV infection (anti-HIV infection therapy) differential equation model with a saturated infection rate. The basic virus reproductive number *R*_0_ of the model is independent of a patient's plasma total CD4^+^ T cell counts *λ*/*d*_1_. This suggests that our model is more reasonable than the model proposed by Srivastava and Chandra in 2010 [13] whose basic virus reproductive number *R*_0_ is dependent on *λ*/*d*_1_ which follows that the more CD4^+^ T cell counts an individual has, the more easily the individual is infected by HIV.
2. This paper proposes and proves two theorems (Theorems 2 and 5) on the globally asymptotical stabilities of the infection-free equilibrium point *Q*_1_ and the endemic infection equilibrium point *Q*_2_ of the modified model.
3. This paper points out the implications of the two theorems which are ignored by other similar researches on modelling HIV infection (anti-HIV infection therapy):
  - A person with the basic virus reproductive number *R*_0_ < 1 will recover automatically even if the person is infected with a large amount of HIV. If a treatment makes a patient's *R*_0_ < 1, this patient will be cured eventually even if infected with a large amount of HIV.
  - A person with *R*_0_ > 1 will have endemic infection even if the person is infected with only one HIV. If a treatment cannot make a patient's *R*_0_ < 1, the patient's HIV RNA in vivo cannot be cleared up eventually.

The recent reports on three HIV infected patients show that HIV infected patients may be cured via bone marrow transplants (e.g., see: Berlin Patient, first person cured of HIV, may soon have company, Los Angeles Times, July 27, 2012; after Berlin Man, two reported cured of HIV in Kenya, Africa Review, May 6, 2013).

These reports can make one postulate that most individuals who connect HIV virus will not be infected by it and are not infecting other people, who will recover automatically without any treatment. In this case, *R*_0_ = 0 where *k*_1_ = *a* = 0. The fact has not been recognized since AIDS has been discovered in 1983.

In the report [35], a small proportion of human immunodeficiency virus type 1 (HIV-1) infected individuals, called elite and viremic controllers, spontaneously control plasma HIV RNA levels to undetectable (elite controller) or <2000 copies/mL (viremic controller) in the absence of antiretroviral therapy.

These phenomena can be interpreted by our Theorem 2: HIV infected people's basic virus reproductive number *R*_0_ < 1.

(4) Based on the simulation results, we can propose the following hypotheses:
  - for a poor HIV treatment response patient, the drug resistance appears when the patient's HIV RNA level reduces to the first lowest level;
  - Once a patient's drug resistance appears, the patient's HIV promotes the apoptosis of CD4^+^ T cells more strongly;
  - According to the 2013 HIV therapy guidelines published by WHO [32], treatment failure is defined by a persistently detectable viral load exceeding 1000 copies/mL after at least six months of using antiretroviral drugs. Our long-term numerical simulation predictions suggest that after ending the 12 weeks' antiretroviral treatment [24], the two group patients' mean HIV RNA levels keep exceeding 1000 copies/mL during the additional 2 years' anti-HIV infection treatment. The treatments for the two groups are failure and better anti-HIV infection therapies should be considered. This means that the additional 2 years' treatments are not able to make patients obtain better outcomes. This may interpret why WHO defines that half a year's treatment cannot suppress a patient's HIV level below 1000 copies/mL to be treatment failure.

## Figures and Tables

**Table 1:** Parameter values and *R*_0_ at different weeks.

Weeks	*d*_1_	*m*	*n*	*R*_0_
0~4	0.033	0.53	0.84	0.6148
4~12	0.038	0.45	0.76	1.0792

**Table 2:** Parameter values and *R*_0_ at different weeks.

Weeks	*d*_1_	*m*	*n*	*R*_0_
0~4	0.041	0.65	0.68	0.6435
4~12	0.0428	0.55	0.59	1.0600
